# Supplementary material for: Primary Processing and Storage Affect the Dominant Microbiota of Fresh and Chill-Stored Sea Bass Products
Source: Foods. 2021 Mar 22;10(3):671. doi: 10.3390/foods10030671 (PMC8004183; doi:10.3390/foods10030671)
Supplement: Supplementary file 1 [file foods-10-00671-s001.pdf]

Supplementary Materials:

Phylotypes

SBS-FS1

TCAGTGTCAGTATCAGTCCAGGTGGTCGCCTTCGCCACTGGTGTTCCTTCCTATATCTACGCATTT
CACCGCTACACAGGAAATTCCACCACCCTCTACCATACTCTAGTCAGTCAGTTTTGAATGCAGT
TCCCAGGTTGAGCCCGGGGATTTCACATCCAACTTAACTAACCACCTACGCGCGCTTTACGCCC
AGTAATTCCGATTAACGCTTGCACCCTCTGTATTACCGCGGCTGCTGGCACAGAGTTAGCCGGT
GCTTATTCTGTCGGTAACGTCAAAATTGCAGAGTATTAATCTACAACCCTTCCTCCCAACTTAAA
GTGCTTTACAATCCGAAGACCTTCTTCACACACGCGGCATGGCTGGATCAGGCTTTCGCCCATT
GTCCAATATTCCCCACTGCAGCC

SBS-FS2

GTCAGTATTAGTCCAGGTGGTCGCCTTCGCCACTGGTGTTCCTTCCTATATCTACGCATTTCACCG
CTACACAGGAAATTCCACCACCCTCTACCATACTCTAGTCAGTCAGTTTTGAATGCAGTTCCCA
GGTTGAGCCCGGGGATTTCACATCCAACTTAACAAACCACCTACGCGCGCTTTACGCCCAGTAA
TTCCGATTAACGCTTGCACCCTCTGTATTACCGCGGCTGCTGGCACAGAGTTAGCCGGTGCTTAT
TCTGTCGGTAACGTCAAAATTGCAGAGTATTAATCTACAACCCTTCCTCCCAACTTAAAGTGCTT
TACAATCCGAAGACCTTCTTCACACACGCGGCATGGCTGGATCAGGCTTTCGCCCATTGTCCAA
TATTCCCCACTGCAG

SBS-FS3

GTCAGTATCAGTCCAGGTGGTCGCCTTCGCCACTGGTGTTCCTTCCTATATCTACGCATTTCACC
GCTACACAGGAAATTCCACCACCCTCTACCATACTCTAGCTTGTCAGTTTTGAATGCAGTTCCCA
GGTTGAGCCCGGGGATTTCACATCCAACTTAACAAACCACCTACGCGCGCTTTACGCCCAGTAA
TTCCGATTAACGCTTGCACCCTCTGTATTACCGCGGCTGCTGGCACAGAGTTAGCCGGTGCTTAT
TCTGTCGGTAACGTCAAAACACTAACGTATTAGGTTAATGCCCTTCCTCCCAACTTAAAGTGCTT
TACAATCCGAAGACCTTCTTCACACACGCGGCATGGCTGGATCAGGCTTTCGCCCATTGTCCAA
TATTCCCCACTG

SBS-FS4

CAGTGTCAGTATCAGTCCAGGTGGTCGCCTTCGCCACTGGTGTTCCTTCCTATATCTACGCATTTC
ACCGCTACACAGGAAATTCCACCACCCTCTACCATACTCTAGCTCGCCAGTTTTGGATGCAGTT
CCCAGGTTGAGCCCGGGGATTTCACATCCAACTTAACGAACCACCTACGCGCGCTTTACGCCCA
GTAATTCCGATTAACGCTTGCACCCTCTGTATTACCGCGGCTGCTGGCACAGAGTTAGCCGGTG
CTTATTCTGTCGGTAACGTCAAAATTGCAGAGTATTAATCTACAACCCTTCCTCCCAACTTAAAG
TGCTTTACAATCCGAAGACCTTCTTCACACACGCGGCATGGCTGGATCAGGCTTTCGCCCATTGT
CCAATATTCCCCACTGC

SBS-FS5

TGTCAGTATCAGTCCAGGTGGTCGCCTTCGCCACTGGTGTTCCTTCCTATATCTACGCATTTCACC
GCTACACAGGAAATTCCACCACCCTCTACCATACTCTAGCTTGCCAGTTTTGGATGCAGTTCCCA
GGTTGAGCCCGGGGATTTCACATTCAACTTAACAAACCACCTACGCGCGCTTTACGCCCAGTAA
TTCCGATTAACGCTTGCACCCTCTGTATTACCGCGGCTGCTGGCACAGAGTTAGCCGGTGCTTAT
TCTGTCGGTAACGTCAAAGCACTAACGTATTAGGTTAATGCCCTTCCTCCCAACTTAAAGTGCTT
TACAATCCGAAGACCTTCTTCACACACGCGGCATGGCTGGATCAGGCTTTCGCCCATTGTCCAA
TATTCCCCACTGCAG

SBS-FS6

GTCAGTCTTTGTCCAGGGGGCCGCCTTCGCCACCGGTATTCCTCCAGATCTCTACGCATTTCACC
GCTACACCTGGAATTCTACCCCCCTCTACAAGACTCTAGTTCGCCAGTTCGAAATGCAATTCCC
AGGTTGAGCCCGGGGCTTTCACATCTCGCTTAACAAACCGCCTGCGCACGCTTTACGCCCAGTA
ATTCCGATTAACGCTCGGACCCTCCGTATTACCGCGGCTGCTGGCACGGAGTTAGCCGGTCCTT
CTTCTGTAGGTAACGTCACAGCAACAGCGTATTAAGCTGCTACCTTTCCTCCCTACTGAAAGTGC
TTTACAACCCGAAGGCCTTCTTCACACACGCGGCATGGCTGCATCAGGGTTTCCCCCATTGTGC
AATATTCCCCACTGCAGC

SBS-FS7
GTCAGTCTTTGTCCAGGGGGCCGCCTTCGCCACCGGTATTCCTCCAGATCTCTACGCATTTCACC
GCTACACCTGGAATTCTACCCCCCTCTACAAGACTCTAGTTCGCCAGTTCAAAATGCAATTCCC
AGGTTGAGCCCGGGGCTTTCACATCTTGCTTAACAAACCGCCTGCGCACGCTTTACGCCCAGTA
ATTCCGATTAACGCTCGGACCCTCCGTATTACCGCGGCTGCTGGCACGGAGTTAGCCGGTCCTT
CTTCTGTAGGTAACGTCACAGTAACGGGCTATTAACACGCTACCTTTCCTCCCTACTGAAAGTGC
TTTACAACCCGAAGGCCTTCTTCACACACGCGGCATGGCTGGATCAGGGTTGCCCCCATTGTCC
AATATTCCCCACTGCAGC

SBS-FS8
GTCAGTCTTTGTCCAGGGGGCCGCCTTCGCCACCGGTATTCCTCCAGATCTCTACGCATTTCACC
GCTACACCTGGAATTCTACCCCCCTCTACAAGACTCTAGTTCGCCAGTTCGAAATGCTATTCCTA
GGTTGAGCCCAGGGCTTTCACATCTCGCTTAACAAACCGCCTGCGCACGCTTTACGCCCAGTAA
TTCCGATTAACGCTCGGACCCTCCGTATTACCGCGGCTGCTGGCACGGAGTTAGCCGGTCCTTCT
TCTGTAGGTAACGTCACAGATATAGCGTATTAAACTACACCCTTTCCTCCCTACTGAAAGTGCTT
TACAACCCGAAGGCCTTCTTCACACACGCGGCATGGCTGCATCAGGGTTTCCCCCATTGTGCAA
TATTCCCCACTGCAGC

SBS-FS9
TCTTTGTCCAGGGGGCCGCCTTCGCCACCGGTATTCCTCCAGATCTCTACGCATTTCACCGCTAC
ACCTGGAATTCTACCCCCCTCTACAAGACTCTAGCTGGACAGTTTTAAATGCAATTCCCAGGTTG
AGCCCGGGGCTTTCACATCTAACTTATCCAACCGCCTGCGTGCGCTTTACGCCCAGTAATTCCG
ATTAACGCTTGCACCCTCCGTATTACCGCGGCTGCTGGCACGGAGTTAGCCGGTGCTTCTTCTGC
GAGTAACGTCACAGTTGATACGTATTAGGCATCAACCTTTCCTCCTCGCTGAAAGTGCTTTACA
ACCCGAAGGCCTTCTTCACACACGCGGCATGGCTGCATCAGGGTTTCCCCCATTGTGCAATATT
CCCCACTGC

SBS-FS10
ACCAGAGAGTCGCCTTCGCCACTGGTGTTCCTCCACATCTCTACGCATTTCACCGCTACACGTGG
AATTCCACTCTCCTCTTCTGCACTCAAGTTCCCCAGTTTCCAATGACCCTCCCCGGTTGAGCCGG
GGGCTTTCACATCAGACTTAAGAAACCGCCTGCGCGCGCTTTACGCCCAATAATTCCGGACAAC
GCTTGCCACCTACGTATTACCGCGGCTGCTGGCACGTAGTTAGCCGTGGCTTTCTGGTTAGGTAC
CGTCAAGGTRCCGCCCTATTCGAACGGTACTTGTTCTTCCCTAACAACAGAGTTTTACGATCCGA
AAACCTTCATCACTCACGCGGCGTTGCTCCGTCAGACTTTCGTCCATTGCGGAAGATTCCCTACT
GC

SBS-FS11
GTCAGTCTTTGTCCAGGGGGCCGCCTTCGCCACCGGTATTCCTCCAGATCTCTACGCATTTCACC
GCTACACCTGGAATTCTACCCCCCTCTACAAGACTCTAGCTTGCCAGTTTCAAATGCAGTTCCCA
CGTTAAGCGCGGGGATTTCACATCTGACTTAACAAACCGCCTGCGTGCGCTTTACGCCCAGTAA
TTCCGATTAACGCTTGCACCCTCCGTATTACCGCGGCTGCTGGCACGGAGTTAGCCGGTGCTTCT
TCTGCGAGTAACGTCAATGAACAGTGCTATTAACACTGAACCCTTCCTCCTCGCTGAAAGTGCT
TTACAACCCGAAGGCCTTCTTCACACACGCGGCATGGCTGCATCAGGCTTGCGCCCATTGTGCA
ATATTCCCCACTGCAG

SBS-FS12
GTCTTTGTCCAGGGGGCCGCCTTCGCCACCGGTATTCCTCCAGATCTCTACGCATTTCACCGCTA
CACCTGGAATTCTACCCCCCTCTACAAGACTCTAGCTTGCCAGTTTCAAATGCAGTTCCCAAGTT
AAGCTCGGGGATTTCACATCTGACTTAACAAACCGCCTGCGTGCGCTTTACGCCCAGTAATTCC
GATTAACGCTTGCACCCTCCGTATTACCGCGGCTGCTGGCACGGAGTTAGCCGGTGCTTCTTCTG
CGAGTAACGTCAATGAACAGTGCTATTAACACTGAACCCTTCCTCCTCGCTGAAAGTGCTTTAC
AACCCGAAGGCCTTCTTCACACACGCGGCATGGCTGCATCAGGCTTGCGCCCATTGTGCAATAT
TCCCCACTGCAG

SBS-FS13
GTCAGTCTTTGTCCAGGGGGCCGCCTTCGCCACCGGTATTCCTCCAGATCTCTACGCATTTCACC
GCTACACCTGGAATTCTACCCCCCTCTACAAGACTCCAGCCTGCCAGTTTCGAATGCAGTTCCC

AGGTTGAGCCCGGGGATTTCACATCCGACTTGACAGACCGCCTGCGTGCGCTTTACGCCCAGTA
ATTCCGATTAACGCTTGCACCCTCCGTATTACCGCGGCTGCTGGCACGGAGTTAGCCGGTGCTTC
TTCTGCGGGTAACGTCAATTGACGAGGTTATTAACCTCATCACCTTCCTCCCCGCTGAAAGTACT
TTACAACCCGAAGGCCTTCTTCATACACGCGGCATGGCTGCATCAGGCTTGCGCCCATTGTGCA
ATATTCCCCACTGCA

SBS-FS14
CAGTGTCAGTATTAGTCCAGGTGGTCGCCTTCGCCACTGGTGTTCCTTCCTATATCTACGCATTTC
ACCGCTACACAGGAAATTCCACCACCCTCTACCATACTCTAGTCAGTCAGTTTTGAATGCAGTT
CCCAGGTTGAGCCCGGGGATTTCACATCCAACTTAACAAACCACCTACGCGCGCTTTACGCCCA
GTAATTCCGATTAACGCTTGCACCCTCTGTATTACCGCGGCTGCTGGCACAGAGTTAGCCGGTG
CTTATTCTGTCGGTAACGTCAAAACACTAACGTATTAGGTTAATGCCCTTCCTCCCAACTTAAAG
TGCTTTACAATCCGAAGACCTTCTTCACACACGCGGCATGGCTGGATCAGGCTTTCGCCCATTGT
CCAATATTCCCCACTGCAGC

SBS-FS15
GTCAGTGTTGGTCCAGGTAGCTGCCTTCGCCATGGATGTTCCTCCTGATCTCTACGCATTTCACT
GCTACACCAGGAATTCCGCTACCCTCTACCACACTCTAGTCGTCCAGTTTCCACTGCAGTTCCCA
GGTTGAGCCCAGGGCTTTCACAACAGACTTAAACAACCACCTACGCACGCTTTACGCCCAGTA
ATTCCGAGTAACGCTTGCACCCTTCGTATTACCGCGGCTGCTGGCACGAAGTTAGCCGGTGCTT
ATTCTTTGGGTACCGTCATCCCAACCGGGTATTAACCAGCTGGATTTCTTTCCCAACAAAAGGG
CTTTACAACCCGAAGGCCTTCTTCACCCACGCGGTATGGCTGGATCAGGCTTGCGCCCATTGTCC
AATATTCCCCACTG

SBS-FS16
CAGTGTCAGTATCAGTCCAGGTAGTCGCCTTCGCCACTGGTGTTCCTTCCTATATCTACGCATTTC
ACCGCTACACAGGAAATTCCACTACCCTCTACCATACTCTAGCTTGCCAGTTTTGGATGCAGTTC
CCAGGTTGAGCCCGGGGATTTCACATTCAACTTAACAAACCACCTACGCGCGCTTTACGCCCAG
TAATTCCGATTAACGCTTGCACCCTCTGTATTACCGCGGCTGCTGGCACAGAGTTAGCCGGTGCT
TATTCTGTCGGTAACGTCAAGACARTCACGTATTAGGTAACTGCCCTTCCTCCCAACTTAAAGTG
CTTTACAATCCGAAGACCTTCTTCACACACGCGGCATGGCTGGATCAGGCTTTCGCCCATTGTCC
AATATTCCCCACTG

SBS-FS17
TCAGCGTCAGTTACAGACCAGAAAGTCGCCTTCGCCACTGGTGTTCCTCCATATCTCTGCGCATT
TCACCGCTACACATGGAATTCCACTTTCCTCTTCTGCACTCAAGTTTTCCAGTTTCCAATGACCCT
CCACGGTTGAGCCGTGGGCTTTCACATCAGACTTAAAAAACCGCCTACGCGCGCTTTACGCCCA
ATAATTCCGGATAACGCTTGCCACCTACGTATTACCGCGGCTGCTGGCACGTAGTTAGCCGTGG
CTTTCTGATCAGGTACCGTCAAGATGTGCACAGTTACTTACACATTTGTTCTTCCCTGATAACAG
AGTTTTACGATCCGAAGACCTTCATCACTCACGCGGCGTTGCTCCGTCAGGCTTTCGCCCATTGC
GGAAGATTCCCTACTGC

SBS-FS18
CGTCAGTCTTTGTCCAGGGGGCCGCCTTCGCCACCGGTATTCCTCCAGATCTCTACGCATTTCAC
CGCTACACCTGGAATTCTACCCCCCTCTACAAGACTCTAGCTTGCCAGTTTCAAATGCAGTTCCC
AAGTTAAGCTCGGGGATTTCACATCTGACTTAACAAACCGCCTGCGTGCGCTTTACGCCCAGTA
ATTCCGATTAACGCTTGCACCCTCCGTATTACCGCGGCTGCTGGCACGGAGTTAGCCGGTGCTTC
TTCTGCGAGTAACGTCAATCGTTGCAGTTATTAACTACAACGCCTTCCTCCTCGCTGAAAGTGCT
TTACAACCCTAAGGCCTTCTTCACACACGCGGCATGGCTGCATCAGGCTTGCGCCCATTGTGCA
ATATTCCCCACTGC

SBS-FS19
GTCAGTCTTTGTCCAGGGGGCCGCCTTCGCCACCGGTATTCCTCCAGATCTCTACGCATTTCACC
GCTACACCTGGAATTCTACCCCCCTCTACAAGACTCTAGCCTGCCAGTTTCGAATGCAGTTCCCA
GGTTGAGCCCGGGGATTTCACATCCGACTTGACAGACCGCCTGCGTGCGCTTTACGCCCAGTAA
TTCCGATTAACGCTTGCACCCTCCGTATTACCGCGGCTGCTGGCACGGAGTTAGCCGGTGCTTCT
TCTGCGGGTAACGTCAATYGATGAGGTTATTAACCTCAACACCTTCCTCCCCGCTGAAAGTACTT

TACAACCCGAAGGCCTTCTTCATACACGCGGCATGGCTGCATCAGGCTTGCGCCCATTGTGCAA
TATTCCCCACTGCA
